# Reducing unscheduled hospital care for adults with diabetes following a hypoglycaemic event: which community-based interventions are most effective? A systematic review

**DOI:** 10.1007/s40200-021-00817-z

**Published:** 2021-06-10

**Authors:** Aoife Watson, Donna McConnell, Vivien Coates

**Affiliations:** Faculty of Life and Health Sciences, School of Nursing, Ulster University, Magee, Northland Road, Derry, BT48 7JL UK

**Keywords:** Diabetes, Intervention, Prehospital, Community care, Reduce unscheduled hospital care

## Abstract

**Aim:**

To determine which community-based interventions are most effective at reducing unscheduled hospital care for hypoglycaemic events in adults with diabetes.

**Methods:**

Medline Ovid, CINAHL Plus and ProQuest Health and Medical Collection were searched using both key search terms and medical subject heading terms (MeSH) to identify potentially relevant studies. Eligible studies were those that involved a community-based intervention to reduce unscheduled admissions in adults with diabetes. Papers were initially screened by the primary researcher and then a secondary reviewer. Relevant data were then extracted from papers that met the inclusion criteria.

**Results:**

The search produced 2226 results, with 1360 duplicates. Of the remaining 866 papers, 198 were deemed appropriate based on titles, 90 were excluded following abstract review. A total of 108 full papers were screened with 19 full papers included in the review. The sample size of the 19 papers ranged from n = 25 to n = 104,000. The average ages within the studies ranged from 41 to 74 years with females comprising 57% of the participants. The following community-based interventions were identified that explored reducing unscheduled hospital care in people with diabetes; telemedicine, education, integrated care pathways, enhanced primary care and care management teams.

**Conclusions:**

This systematic review shows that a range of community-based interventions, requiring different levels of infrastructure, are effective in reducing unscheduled hospital care for hypoglycaemia in people with diabetes. Investment in effective community-based interventions such as integrated care and patient education must be a priority to shift the balance of care from secondary to primary care, thereby reducing hospital admissions.

**Supplementary Information:**

The online version contains supplementary material available at 10.1007/s40200-021-00817-z.

## Introduction

Diabetes is a complex, chronic condition that requires a high level of self-management in order to maintain normal blood glucose levels as well as continual monitoring from healthcare professionals [1]. Globally, it is estimated over 463 million people live with diabetes [2]. Achieving normal blood glucose levels is hard for many people due to lack of support, increasing age, poor education and poor medication concordance and unexpected activities/events which can lead to numerous consequences, both short term and long term, particularly in urgent situations [3]. In the short term, irregular blood glucose levels can lead to an increase in the severity of high and low glucose levels, potentially resulting in unscheduled care. Whilst in the long term they can result in macrovascular complications such as heart disease, stroke and limb amputations, as well as microvascular complications such as retinopathy, neuropathy and nephropathy [4–6]. These complications can be exacerbated by a lack of knowledge of what are satisfactory blood glucose levels, inadequate management of the treatment regimes, lifestyle challenges and psychosocial and/ or emotional problems [7].

People with diabetes may require unscheduled hospital care for a variety of unavoidable medical emergencies including stroke, myocardial infarction, trauma and loss of consciousness whereby hospital care is vital [8]. However, unscheduled hospital care be avoided for acute complications such as hypoglycaemia and hyperglycaemia if adequate community care is received [9]. Unscheduled hospital care refers to any healthcare that is unplanned, including prehospital care, emergency department (ED) care, specialist hospital support or admissions and hospitalisations [10]. A prehospital setting refers to any care that is received by a patient in the community prior to their arrival in hospital. In 2016 in the USA, there were 16 million visits to hospital by adults whereby a diagnosis for diabetes was listed. Of these visits, 224,000 were for hyperglycaemia, 203,000 were for diabetic ketoacidosis and 235,000 were for hypoglycaemia [11]. Severe hypoglycaemia has been associated with greater blood glucose variability and higher haemoglobin A1c (HbA1c) levels, making it harder to manage and thus requiring more ambulance call outs [12]. Severe hypoglycaemic events (SHE) account for 48,000–98,400 emergency ambulance calls within Scotland and England annually [13].

Diabetes costs the NHS over £3bn per year, accounting for approximately 10% of the budget [14]. Some of these costs arise from ambulance calls that result in conveying a person to hospital which costs approximately £359.51 per patient, with non- elective admissions in England for people with diabetes costing over £1.6bn annually, albeit not all for diabetic specific causes [12, 15]. In addition to the direct costs relating to diabetes, there are also indirect costs and care burdens that arise including loss of earnings from time off work and the need for informal care [16].

Reducing unscheduled admissions for diabetes has the potential to reduce costs on the health services by providing the right care, in the right place, at the right time, by placing the patient at the centre of the model which aligns with the Transforming Your Care (TYC) strategy in the UK [45]. Fewer unscheduled admissions could also reduce overcrowding and clinical pressures in the emergency department, leading to reduced waiting times.

A range of interventions have been trialled to reduce unscheduled admissions in people with diabetes; from treat and leave protocols, to telemedicine, to integration of care between primary care providers and specialists. The objective of this systematic review is to determine which community-based interventions are most effective at reducing unscheduled hospital care for hypoglycaemic events in adults with diabetes. To date, there have been no other published systematic reviews conducted investigating this topic, despite the importance and implications for clinical practice and research.

## Methods

### Protocol and registration

The protocol for this review was registered in the International Prospective Register of Systematic Reviews (PROSPERO) [CRD42019132649]. The review was carried out in accordance with the Preferred Reporting Items for Systematic Reviews and Meta-Analysis (PRISMA) guidelines. See supplementary file (PRISMA 2020 checklist).

### Search strategy

**Fig. 1:**
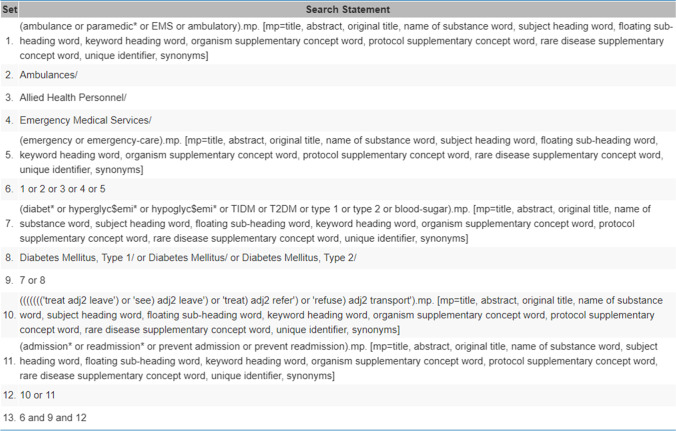
Screenshot of search used in Medline Ovid

The search strategy was developed alongside the subject librarian and confirmed with the project team, with the final search taking place on 8th April 2020. Three databases; Medline Ovid, CINAHL Plus and ProQuest Health and Medical Collection were systematically searched, with EMBASE and AMED included in the Medline Ovid database. The search was developed for Medline Ovid and adapted for the other databases as seen in Fig. 1. Both key search terms and medical subject heading terms (MeSH)/ Thesaurus terms were used to identify relevant publications from January 2014 to April 2020. The lower year limit of 2014 was selected to focus on recent publications due to the rapid progression of healthcare and technology. A search of the reference lists in relevant papers was also carried out to identify all relevant papers to determine which interventions were effective at reducing unscheduled hospital care in people with diabetes. The key search strategy and key words were: (diabet* or hypoglyc$emi* or TIDM or T2DM or type 1 or type 2 or blood-sugar or Diabetes Mellitus, Type 1/ or Diabetes Mellitus/ or Diabetes Mellitus, Type 2/) AND (ambulance or paramedic* or EMS or ambulatory or Ambulances/ or Allied Health Personnel/ or Emergency Medical Services/ or emergency or emergency-care) AND ('treat adj2 leave') or 'see) adj2 leave') or 'treat) adj2 refer') or 'refuse) adj2 transport' or admission* or readmission* or prevent admission or prevent readmission). A hand search of the grey literature and the reference lists in relevant papers was also carried out to identify all relevant papers to determine which interventions were effective at reducing unscheduled care in people with diabetes.

### Selection criteria

The inclusion and exclusion criteria were developed alongside the review question using the PICOS (participants, interventions, comparisons, outcomes and study design) strategy.

Papers were initially screened based upon their titles and abstract. The inclusion criteria were that the paper related to people with (1) diabetes, (2) participants were over 18 years old, (3) included an intervention and (4) was undertaken in a prehospital community setting. Eligible papers were then screened in full to determine whether they (5) addressed reducing unscheduled hospital care. Papers were excluded from the search if they were not written in English, did not involve human subjects and/or were not published since 2014. A sample of papers were then reviewed by a second reviewer (VC) who was blinded from the prior decision and any disagreements discussed until resolved. A third reviewer (DMcC) was consulted on any papers where an agreement was not reached.

### Data extraction

For papers that met the inclusion criteria, data were extracted by one reviewer (AW) and inputted into a form in Microsoft Excel. Data extraction was guided by the PICOS strategy and involved; study identification (title, first author, year), location of study, description of intervention, category of intervention, length of time of intervention, study design, SURE guideline used and score, sample size, percentage of participants that were female, average age, study outcomes, limitations and any notes on the study. Authors were contacted if further information or clarification was needed. If this information remained unavailable, the data cell was left blank. Any papers that were missing data on the type of intervention or study outcome were excluded if the author could not be contacted for clarification.

### Quality assurance

The quality of the studies was assessed using the Specialist Unit for Review Evidence (SURE) critical appraisal guidelines to critique health related research and identify the ways errors and bias can distort research results, tailored to the relevant study design [17, 18]. When completing the checklist, 1 point was assigned for ‘Yes’ answers, with 0 points assigned for ‘No’ or ‘Can’t tell’ answers. The total points were added up and then converted to percentages to class the studies as high, moderate or poor quality. Studies were described as ‘high’ quality if they scored > 80%, ‘moderate’ quality if they scored > 60 < 80% and ‘poor’ quality if they scored < 60%. Papers were reviewed for quality by a second reviewer (VC) for rigour and any disagreements discussed. A third reviewer (DMcC) was consulted on any papers where an agreement was not reached.

## Results

### Summary

**Fig. 2:**
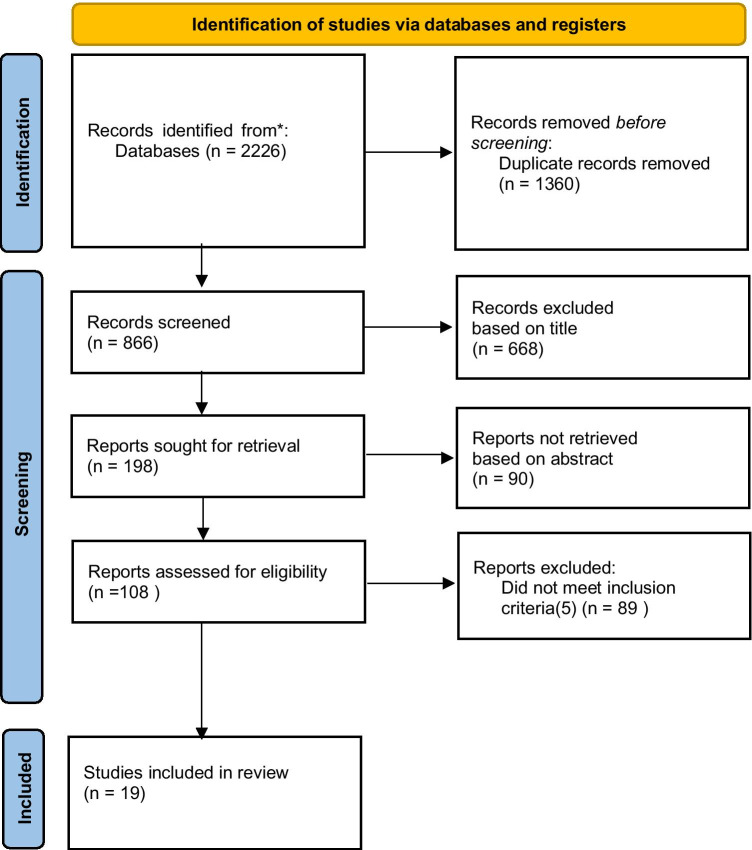
Summary of paper selection from PRISMA flow diagram

The study selection process is outlined in Fig. 2. The search produced 866 unique citations that were screened based on their title and abstract with 108 full texts requested. There were 19 papers that met the full inclusion criteria and had sufficient data to be included in the analysis. These papers are summarised in Table 1.

**Table 1:** Summary of paper characteristics used in review

Author	Year of Publication	Location	Intervention	Length of Time	Category of Intervention	Study Design	SURE	Quality	Sample	Males	Females	Mean Age	Finding
							Guideline Used	Rating	Size				
Kaufman et al.	2014	USA	High risk patients reached out to by care management teams composed of registered nurse, licensed practice nurse, community health worker, health coaches, social work staff	90 day intervention with 6 month follow up	Care	Unclear	Study design	NA	25	36%	64%	63	Reduction in hospital admissions.
					management		unclear- not completed						
					intervention								
Kearns et al.	2017	USA	Care management teams were assigned to intervention practices composed of a physician, care manager, medical assistant	3 years	Care management team	Randomised prospective cohort study	RCT	Moderate	19,696	48%	52%	55	ER visits and readmissions increased while hospital admissions and urgent care visits decreased across both groups.
Ginzburg et al.	2017	Israel	Nurse managed care management team composed of physician, nurse, social worker, pharmacist, physical exercise consultant, other medical specialists	6 months	Care management team	Retrospective cross sectional study	Cross sectional	Low	100	52%	48%	63	Decrease in hospitalisations. Increase in dietician, ophthalmologist, family physician visits
Brophy et al.	2014	USA	Collaborative drug therapy management providing health coaching, education, transportation assistance, prescription refills	1 year	Drug therapy	Retrospective quasi experiment with comparison group	RCT	Moderate	1764	45%	55%	58	Reduction in hospital admissions (significant KF group, non significant ACP group), reduction in ED visits.
Yeung et al.	2014	USA	6 month low intensity, 24 month high intensity self management and support groups by certified diabetes educator and clinical psychologist	2.5 years	Education	Single cohort, time series prospective study	Cohort	Moderate	60	30%	70%	62	No significant difference in acute care use, non acute care use, days lost to disability found. Increase in non acute care use approached significance. Decrease in urgent care and ED visits. Improvement in clinical markers and self care.
Elliott et al.	2014	UK	DAFNE	5 day course	Education	Retrospective observational study	Study design	NA	939	53%	47%	41	Significant reduction in ketoacidosis episodes, hypoglycaemia episodes, hospital admissions, ED visits, paramedic call outs. Improvements in glycaemic control and quality of life.
							doesn't fit- not completed						
Wong et al.	2014	Hong Kong	Patient empowerment programme giving patients greater control over their health through collaboration with the healthcare provdider	12 month follow up	Enhanced	Observational, matched, cohort study	Cohort	High	2282	50%	50%	64	Significantly improved clinical outcomes in HvA1c, LDL-C, improved clinical outcomes in BP. Significant reductions in GOPC vivits, reduction in SOPC visits, ED visits, inpatient admissions
					primary care program								
MacKay et al.	2014	Canada	Modified role of medical office assistant to do key tasks for people with diabetes	12 months	Enhanced	Unclear	Study design	NA	96	47%	53%	66	Similar number of patients with ED visits and hospital admissions
					primary care program		unclear- not completed						
Seidu et al.	2016	UK	Enhanced practices used primary care physicians (PCPs) with an interest in diabetes, supported by multidisciplinary primary care teams to provide care to patients dischardged from secondary care in primary care	12 month follow up	Enhanced	Before and after	Case Control	High	8366	50%	50%	Unknown	Decrease in admissions and outpatient attendances.
					primary care program								
Peterson et al.	2017	USA	Extension of CareFirst's program to Medicare. Nurses worked with patients’ usual primary care practitioners to coordinate care for high-risk Medicare patients.	2.5 years	Enhanced	Unclear	Study design	NA	104,000	41%	59%	74	Decline in hospitalisation rates by 10% in intervention group, similar to control group.
					primary care program		unclear- not completed						
Goff et al.	2018	USA	Team based care model using 2 registered nurses, 2 medical assistance trained as outreach workers, case manager	12 month follow up	Enhanced	Controlled before and after observational study	Case Control	High	319	36%	64%	53	Reduction in unplanned
					primary care program								hospitalisation rates, no change in annual rate of ED visits.
Zurovac et al.	2019	USA	Behavioural health integrated into primary care with physicians, nurses, medical assistants, practice manager, behavioural health therapists, registered nurse health coaches, panel manager	24 months	Enhanced	Observational study	Study design	NA	2001	41%	59%	72	No significant reduction in hospitalisations. Reduced ED visits. Improvement in diabetes care
					primary care program		doesn't fit- not completed						
McLendon et al.	2019	USA	Care coordination, telemedicine, education	12 months	Enhanced	Retrospective study	Study design	NA	59	20%	80%	Unknown	Statistically significant reduction in ED visits, hospital admissions, statistically significant increase in A1c, increased DSME score
					primary care program		doesn't fit- not completed						
Chung et al.	2014	USA	Clinical pharmacy program following collaborative drug therapy management protocol providing 30 minute visits with pharmacist whenever needed	30 minute	Integrated care pathway	Retrospective cohort study	Cohort	High	782	45%	55%	51	Statistically significant reduction in hospitalisations, non significant increase in ED visits, significant reduction in HbA1c levels.
				visit with									
				pharmacist whenever needed with 1 year follow up									
Sampson et al.	2017	UK	Severe hypogylcaemic ambulance management team who referred callers to an education teamthey where then had direct face to face or telephone contact education on SH management and avoidance	17 months	Integrated care pathway	Retrospective study	Study design doesn't fit- not completed	NA	2000	56%	44%	67	Reduction in ambulance transport to hospital and hospital
													admissions
Bennett et al.	2018	USA	Community paramedicine- implementing a care plan devised by liason nurse over a number of visits	Unknown	Integrated care pathway	Pre and post test evaluation with comparison group	Case Control	High	68	40%	60%	58	Significant decrease in ED visits, ambulance calls, inpatient length of stay, hospitalisations, readmisstion rate and increase in transports compared to comparison group.
													Increase in comparison group.
Quan et al.	2015	USA	Automated telephone calls with follow up from health staff/ lay person for behavioural action plan	6 month intervention	Telemedicine	Controlled quasi experimental evaluation trial	RCT	High	362	29%	71%	55	Reduction in ED visits and hospitalizations.
Due-	2015	Denmark	Diabetic specialist nurse answers calls out of hours	6 months	Telemedicine	Sequential exploratory mixed methods study. Observational study	Study design	NA	592	44%	56%	62	ATC prevented the escalation of severe diabetes related conditions and likely prevented admissions in 15/17 cases.
Christensen et al.							doesn't fit- not completed						
Warren et al.	2017	Australia	Care coordinator provided additional assistance with	6 months	Telemedicine	Randomised	RCT	High	126	54%	46%	61	Decrease in GP visits, specialist referrals, hospital admissions.
						control trial							

### Study characteristics and participants

Studies that were included were those published since 2014, with data collected from USA (n = 11) [19–29], UK (n = 3) [30–32], Hong Kong (n = 1) [33], Denmark (n = 1) [34], Canada (n = 1) [35], Israel (n = 1) [36] and Australia (n = 1) [37]. There were a range of interventions identified that explored reducing unscheduled hospital care in people with diabetes; telemedicine (n = 3) [20, 34, 37], education (n = 2) [19, 30], integrated care pathways (n = 4) [23–25, 31], enhanced primary care (n = 7) [26–29, 32, 33, 35] and care management teams (n = 3) [21, 22, 36]. The study design of the papers were heterogenous; 3 before- after studies [25, 26, 32], 1 randomised controlled trial (RCT) [37], 4 cohort studies [19, 22, 23, 33], 2 quasi experiments [20, 24], 1 cross sectional study [36], 1 mixed method study [34], 4 observational or retrospective studies [28–31] and 3 designs were unspecified/unknown [21, 27, 35]. The sample size of the 19 papers ranged from n = 25 to n = 104,000. The average ages within the studies ranged from 41 to 74 years with females comprising 57% of the participants.

### Study quality

Based on the SURE guidelines, there were 7 high quality papers [20, 23, 25, 26, 32, 33, 37], 3 moderate quality papers [19, 22, 24] and 1 low quality paper [36]. It was not possible to complete a critical appraisal for 8 papers; 3 papers where the study design was not clear [21, 27, 35] and 5 papers where the study design did not fit the SURE guidelines (18, 26–28, 31).

### Study outcomes

The papers produced results about hospitalisation rates, with varying degrees of significance. Three papers reported a significant decrease in hospitalisations [23, 24, 30], 12 papers reported a decrease in hospitalisations, although not significant [20, 21, 25–27, 31–37] and 3 reported no change in hospitalisations [19, 22, 28]. No papers reported an increase in hospitalisations due to the intervention.

## Discussion

### Main findings

This systematic review has shown that there are a number of studies relating to interventions that reduce unscheduled hospital care for hypoglycaemic events for adults with diabetes. This is currently particularly important as adults with diabetes have been reluctant to go to hospital during the COVID-19 pandemic, fearful of contracting the virus and consequently missing treatment [38]. The interventions were categorised as telemedicine, education, integrated care pathways, enhanced primary care and care management teams and had varying levels of effectiveness as outlined below.

### Telemedicine

Telemedicine is the delivery of health care at a distance to optimize and improve health outcomes [39]. There were 3 studies that utilised telemedicine, albeit in different ways. Quan et al. used automated telephone calls with behavioural follow ups from health care staff or a trained lay person for 6 months [20]. Due-Christensen et al. utilised acute telephone counselling from a diabetic specialist nurse (DSN) out of hours over 6 months [34]. Warren et al. used a home monitor that captured clinical measurements and provided additional care from a diabetes care coordinator [37].

The use of telemedicine showed a decrease in unscheduled admissions and ED visits across all 3 studies although the results were not significant [20, 34, 37]. These studies were based in Australia [37], USA [20] and Denmark [34], showing success in different countries. Both Warren et al. and Quan et al. used a RCT design to compare efficacy [20, 37] whilst Due- Christensen et al. used an observational design thus not allowing for the control of variables [34]. Whilst there were no significant differences in the findings, a larger sample size might have shown significance as there were only 126 participants in [37], 362 in [20] and 592 in [34].

### Patient education

Patient education is offered to people with long term conditions to aid and enhance their self-management of their health and wellbeing [40]. Elliott et al. evaluated the DAFNE (Dose Adjustment For Normal Eating) education course that is used in the UK and Ireland whilst Yeung et al. developed a 2.5 year long-term education program that provided low intensity self- management education for 6 months before a 24 month high intensity self-management support component with a certified diabetes educator and clinical psychologist [19, 30].

Patient education also showed a decrease in the number of unscheduled admissions and ED visits. Elliott et al. found that this decrease in ED visits and unscheduled admissions was significant [30], however Yeung et al. did not find the decrease in admissions to be significant, nor the reduction in ED visits [19]. This could be due to the small sample size in the study by Yeung et al. [19] (n = 60) and the older age of the participants in the study by Yeung et al. [19] (mean age 62 years old) compared with the study by Elliott et al. [30] (mean age 41 years old). The trials took place in the USA [19] and UK [30]. These results show that a 2.5 year, long- term educational program does not necessarily provide increased benefits over shorter structured education programs.

### Care management teams

Care management teams are a team based approach to helping patients and their support system manage chronic illnesses more effectively [41]. Kaufman et al. used coordinated care management teams composed of a registered nurse, licensed practical nurse, community health worker, health coaches, social work staff, program manager, nurse care manager, program director and associate clinical director to improve care [21]. Ginzburg et al. used a nurse managed care team comprising a physician, nurse, social worker, pharmacist, physical exercise consultant and other medical specialists to achieve optimal diabetes control and management, with telephone reminders included [36]. Kearns et al. compared resource utilization in traditional care with resource utilization in care management teams comprising a physician, medical assistant and care manager, who was a certified diabetes educator [22].

The use of care management teams had mixed results on their efficacy in reducing unscheduled admissions. Kaufman et al. found a decrease in healthcare utilization in both ED visits and admissions however they had a small sample size (n = 25) [21]. Ginzburg et al. also had a relatively small sample size (n = 100). Their study showed a significant increase in non- acute care visits to physicians and ophthalmologists along with a non- significant increase in dietitian visits. These increases, could explain the decrease in hospitalisations observed [36]. Kearns et al. had the largest sample size (n = 19,696), however found no change in healthcare utilization and admissions relevant to the intervention. There was a decrease in urgent care visits and hospital admissions and an increase in ED visits and readmissions, however these changes were seen in both the intervention and control groups, suggesting influence from outside factors not related to the intervention [22].

### Integrated care

Integrated care is the coordination and integration of health services, to ensure the best patient care [42]. The integrated care studies were broken down into those that involved pharmacists and those that utilised the ambulance service. Brophy et al. used collaborative drug management therapy involving both a pharmacist and care manager for high risk patients treated with polypharmacy, providing health coaching, education, transportation assistance and prescription refill assistance [24]. The study by Chung et al. used a clinical pharmacy program under a collaborative drug therapy management program enabling patients to receive a 30 min visit with the pharmacist, when needed, to maintain patient safety and achieve the patients’ goals [23]. Sampson et al. implemented a new integrated care pathway for managing severe hypoglycaemia that involved providing patients with written information on avoiding severe hypoglycaemia and a diabetes education follow up session with an educator within 3 days of their call, unless the patient actively opted out [31]. Bennett et al. used community paramedicine to shift care from ED and inpatients to outpatient and medical home based care by community paramedics implementing a care plan devised by a liaison nurse over a number of visits [25]. Whilst ambulance care is considered unscheduled care, these studies were relevant as they were aimed to prevent hospital admissions and ED attendances.

The pharmacy interventions by both Brophy et al. and Chung et al. showed a statistically significant decrease in hospitalisations upon their implementation. Brophy et al. found that there was a decrease in ED visits although it was not significant [24]. This was contradicted by Chung et al. who found that there was an increase in ED visits, however this increase was less in the intervention group than the comparison group [23]. Whilst both Chung et al. and Brophy et al. had large sample size [23, 24], the sample size in the control and intervention groups in [23] were unevenly matched (557:225) [13]. These studies both used retrospective data analysis and so confounding variables would not have been controlled.

Studies by both Sampson et al. and Bennett et al. involving ambulance services showed a decrease in unscheduled admissions [25, 31]. Bennett et al. showed a decrease in ED visits and ambulance calls, however an increase in those requiring hospital transport for higher levels of care. The control group also reflected these outcomes, however at a lower rate than the intervention group, indicating that the intervention group utilised care more appropriately than the control group [25]. This contradicts the study by Sampson et al. who showed a decrease in hospital transports upon implementation of the new clinical pathway [31].

These studies suggest that better co-ordination across health care sectors and professional groups has the potential to, and has been shown to, improve outcomes in adults with diabetes.

### Enhanced primary care

Enhanced primary care is increased clinical and social support in the community provided by nurses, care coordinators, support workers and others who work alongside GP’s to help patients learn more about and improve their condition management [43]. Zurovak et al. used integrated teams of physicians, nurses, medical assistants, practice managers, behavioural health therapists, registered nurse health coaches and panel managers to provide patient-centred care to improve behavioural health, care management of chronic illnesses and improved technology [28]. MacKay et al. enhanced the role of the medical office assistant to carry out key tasks in diabetes care to find out if it improved the effectiveness of care [35]. The study by Seidu et al. compared practices providing enhanced primary care with practices providing core care. Enhanced practices had a primary care physician and practice nurse with an interest in diabetes who identified patients who could be discharged from secondary care and managed in primary care with monthly meetings discussing care for the complex cases [32]. Wong et al. implemented a patient empowerment program to give patients greater control over their health care decisions utilizing a collaborative approach between the patient and healthcare provider [33]. In Goff et al. ’s study they implemented a team care model, consisting of 2 registered nurses, 2 medical assistants trained as outreach workers and a case manager and compared the outcome and resource utilization with matched controls who did not receive the enhanced care [26]. The study by Peterson et al. utilised nurses to work with the patient and their primary care physician to develop and implement care plans, contacting patients approximately once a week, to measure association with extending CareFirst’s BlueCross BlueShield commercial health insurance program to Medicare Fee-for-service patients on outcome and resource use [27]. Multiple interventions were used by McLendon et al. to enhance diabetes care including nurse care management involving doctors, physician assistants and nurses, telemedicine and education [29].

Enhanced primary care was the most common intervention identified in this review with six studies focused on it. Three studies were based in the USA [26–28], one in the UK [32], one in Hong Kong [33] and one in Canada [35]. Zurovac et al. found a slight, non- significant increase in the number of admissions for ambulatory care sensitive conditions but no overall change in hospitalisations [28]. This contradicted the other five papers that showed a decrease in the number of unscheduled admissions, albeit all non- significantly. MacKay et al. found that whilst there was a decrease in admissions, the control group also had a decrease suggesting external factors not related to the intervention were at play [35]. ED visits were found to be decreased or remained similar in four of the six studies. Seidu et al. did not measure ED visits but commented that they would be unlikely to increase [32] and Zurovac et al. showed an increase in ED visits although at a lower rate than the control, despite the program not employing specific strategies to reduce ED visits [28]. Wong et al. showed an increase in specialist outpatient clinic visits but a significant decrease in general outpatient clinic visits [33] with Seidu et al. showing a decrease in the number of non- elective bed days [32].

Reducing unscheduled admissions for diabetes will help reduce overcrowding and clinical pressures in the emergency department, leading to reduced waiting times. This will also reduce costs on the health services by providing the right care, in the right place, at the right time, by placing the patient at the centre of the model which aligns with the Transforming Your Care (TYC) strategy in the UK [45]. In addition, better co-ordination across health care sectors and professional groups has the potential to, and has been shown to, improve outcomes in adults with diabetes.

## Limitations

There were a number of limitations in relation to this review; the search only included papers written in English and published since 2014 so there could be interventions that pre- dated this or interventions published in other languages not identified in the search. However, as health care and technology progresses so rapidly it was felt important to focus on recent practice as far as possible. The most common limitation that appeared in the studies was an underpowered or small sample size, potentially leading to Type II errors or bias [19, 20, 25, 29, 35–37, 44]. In one study financial incentives were also used to encourage participation which could have produced results that would have not been seen otherwise [27]. Three studies lacked a control group [19, 30, 34] and a number of studies did not blind either participants, healthcare professionals or researchers in the allocation of the intervention arm, potentially leading to bias [20, 24–26, 28–32, 37]. Three studies also had unclear study designs [21, 27, 35]. There were only 7 high quality papers and 3 moderate quality papers, with 1 low quality paper and 8 papers of unknown quality, therefore the strength of the evidence should be considered with caution.

Data extraction was undertaken by one reviewer only (AW) due to the lack of ambiguity in the data to be extracted, having a second reviewer may have added to the rigour of the study.

## Conclusion

The findings in this paper show that globally, there is a scarcity of high-quality research into interventions that reduce unscheduled hospital care in adults with diabetes, despite this being such an important aspect of health care provision. Statistically significant decreases were reported from one study using patient education and two studies promoting integrated care suggesting these are the most effective interventions at reducing unscheduled hospital care for hypoglycaemic events in adults with diabetes. It is evident that there are opportunities to improve integrated care for people with diabetes however, as the quality of the existing evidence base is variable, further high-quality research with larger samples should be carried out to enhance the evidence base around these interventions. Investment in effective community-based interventions must be a priority to shift the balance of care from secondary to primary care to facilitate reduction in unscheduled hospital admissions.

## Electronic supplementary material

Below is the link to the electronic supplementary material.

Supplementary file1 (PDF 144 KB)
